# Phenotypic and functional comparisons between cryopreserved and freshly isolated peripheral blood mononuclear cells with or without red blood cell lysate (ACK) treatment with special focus on regulatory T cells

**DOI:** 10.1177/09636897251382315

**Published:** 2025-10-29

**Authors:** Keyvan Habibi, Nils Ågren, Kaoru Okada, Heléne Johansson, Ming Yao, Makiko Kumagai-Braesch

**Affiliations:** 1 Transplantation Surgery, CLINTEC, Karolinska Institutet, Stockholm, Sweden; 2 ME Transplantation, Karolinska University Hospital, Stockholm, Sweden

**Keywords:** PBMCs, regulatory T cells, cryopreservation, red blood cell lysis, transplantation tolerance

## Abstract

Adaptive transfer of autologous regulatory T cells (Treg), or *ex vivo*-generated immunomodulatory cells, has shown promise in reducing/withdrawing immunosuppression after organ transplantation. The effect of cryopreserving such cells is still unclear. This study aims to evaluate the effects of cryopreservation on the immunomodulatory functions of peripheral blood mononuclear cells (PBMCs) with or without pretreatment with red blood cell (RBC) lysate (ACK). Human PBMCs enriched from buffy coats of healthy blood donors were treated either with ACK or phosphate-buffered saline (PBS). Thereafter, a batch of the PBS-control subset was cryopreserved with 10% dimethyl sulfoxide (DMSO) and subsequently examined for phenotype, functionality, and relative gene expression. We found that ACK-treated PBMCs exhibited higher numbers of interferon gamma (IFN-γ)-producing cells when stimulated with viral peptides (*p* = 0.0078), indicating that ACK treatment may improve the antigen sensitivity of memory T cells. After cryopreservation, contaminated RBCs and granulocytes, cell viability, and CD4^+^ T-cell population decreased (*p* = 0.0078); IL-1β expression increased; and FoxP3 expression decreased (*p* = 0.0312), where the Treg population remained otherwise unchanged. Enriched Tregs from both fresh and frozen PBMCs suppressed the proliferation of anti-CD3/CD28-antibody-stimulated PBMCs equally. In conclusion, the preservation of Treg function following cryopreservation highlights its potential utility in tolerance-induction trials, providing experimental flexibility and simplified logistics.

## Graphical abstract

**Graphical Abstract:**
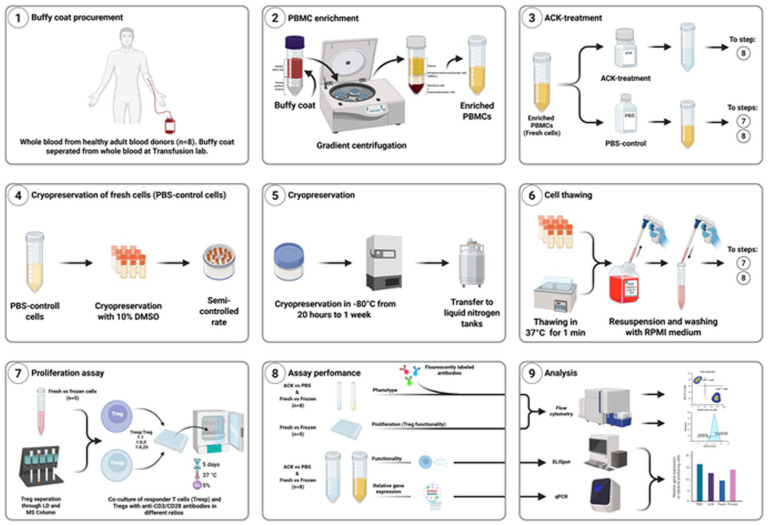

## Introduction

Immunosuppression (IS) is an essential part in maintaining allograft survival and decreasing the risk of rejection. However, IS is associated with several long-term side effects, such as an increased risk of infections, *de novo* malignancies, nephrotoxicity, and cardiovascular and metabolic alterations, for example, hypertension, hyperlipidemia, as well as diabetes mellitus^1–7^.

Advanced cell therapy (ACT) such as regulatory T cells (Treg) or *ex vivo* generated donor-specific anergic cells have been proposed and shown promise in inducing a state of immunological tolerance toward the allograft and thereby avoiding and/or decreasing the need for IS. After promising trials using non-human primates^8^, cell therapy has also been evaluated in clinical phase I/IIa studies and shown to be safe and demonstrated efficacy in tolerance induction^9–11^.

In order to achieve sufficient cell amounts for clinical use, leukapheresis is likely the best option^12^. Leukapheresis separates blood components by density through the use of the centrifugal force and can be tuned to collect peripheral blood mononuclear cell (PBMC)-rich portions and then circulate the rest of the blood components back to the blood donor. The color of the separated portion is an indicator of how rich it is in PBMCs. A light pink color is usually aimed for to balance between efficiency and purity, but a certain amount of RBC contamination is unavoidable, which may affect cell counting and the development of specific T-cell responses. One way to circumvent this issue is to introduce a lysing buffer to the cell product, which we assessed here by using ammonium-chloride-potassium (ACK) solution.

Transplantation surgery is contingent on the availability of donor allografts, with a substantial number of transplantations from deceased donors occurring outside of regular working hours, at which time leukapheresis and intricate cell culture protocols may be difficult to arrange. Cryopreservation of recipient PBMCs in advance could make ACTs more feasible.

There is currently no established consensus on the effect of cryopreservation on PBMCs regarding phenotype and function, and there are few to no studies on the effect of ACK on PBMCs. In this study, we aim to investigate whether cryopreservation affects the phenotype, functionality, and relative gene expression of PBMCs with a special focus on Tregs and their immunosuppressive capacities after cryopreservation. We also aim to determine if ACK influences PBMC function with the same endpoints as cryopreservation.

## Material and methods

### Cell separation

Human PBMCs were enriched from buffy coats from healthy adult blood donors (n = 8) that were obtained from the Department of Transfusion Medicine at Karolinska University Hospital, Huddinge, Sweden, and were processed within 24 h of procurement. As an anticoagulant, acid-citrate-dextrose (ACD-A) solution was used. The only donor information provided was the blood group and that they had not consumed acetylsalicylic acid.

The plasma and cell portions were separated by centrifugation at 400 × *g* for 10 min. The cell portion was diluted with phosphate-buffered saline (PBS, purchased from Karolinska University Hospital, Huddinge, Sweden) up to three times and used for PBMC separation. Fifteen milliliters of Lymphoprep (Stemcell Technologies Inc., Vancouver, Canada) was added to 50-mL SepMate PBMC isolation tubes (Stemcell Technologies), and then 30 mL of the cell suspension was gently layered on top of the Lymphoprep. After centrifugation at 1200 × *g* for 10 min with brake, the PBMC-enriched cell portion was collected and washed with PBS and centrifuged at 350 × *g* for 10 min according to the company’s protocol (Stemcell Technologies). These gradient-separated PBMC portions inevitably contained varying amounts of RBCs and granulocytes; thus, we refer to this preparation as enriched PBMCs in this paper. Collected cell fractions were divided into two groups: (1) red blood cell (RBC) lysate, ACK-treated group, and (2) control PBS-treated group. The cell pellets of the ACK-treated group were suspended in 5-mL ACK (ThermoFisher Scientific, MA, USA); the cells in the control PBS-treated group were suspended in 5-mL PBS. Both groups of cells were incubated for 10 min at room temperature. Cells were then washed three times with PBS and resuspended in 5 mL of Roswell Park Memorial Institute (RPMI) medium (ThermoFisher Scientific) supplemented with 5% fetal bovine serum (FBS) (ThermoFisher Scientific). We opted to use FBS rather than autologous plasma because of the risk of cross-reaction of human heterophilic antibodies against mouse antibodies. Cell concentration and viability were assessed with Solution 13, containing acridine orange (30 μL/mL, DAPI 100 μL/mL; Chemometec, Lillerød, Denmark), and analyzed by NucleoCounter NC-3000 (Chemometec).

### Cryopreservation

For the laboratory tests, such as flowcytometry, ELISpot assays, and quantitative polymerase chain reaction (qPCR), PBMC-enriched cells were suspended with FBS at a concentration of less than 100 million cells/mL. Cryopreservation buffer of PBS solution with 20% dimethyl sulfoxide (DMSO) (ThermoFisher Scientific) was prepared and kept on ice. The same volume of cell suspension and cryobuffer was mixed (final concentration of cells < 50 × 10^6^/mL in 10% DMSO). The 1-mL cell suspension was then aliquoted to 1.8 mL cryotubes, and the tubes were then put into a −80°C freezer for more than 24 h and later maintained in liquid nitrogen tanks until use (−196°C). For functional Treg analysis, cells were cryopreserved in PBS containing 10% human serum albumin and 10% DMSO in order to examine Treg functionality more closely to a clinical setting. This cryopreservation protocol is used for the clinical immunomodulatory cell project (M217) in the GMP laboratory at Karolinska University Hospital, Huddinge, Sweden. The cell concentration was assessed as described earlier. Cells were cryopreserved at a semi-controlled rate in Corning CoolCell Freezing Containers at −80°C from 20 h to 1 week and then transferred to liquid nitrogen tanks.

### Cell thawing

RPMI medium with 5% FBS was prepared. The cryopreserved cells were retrieved from liquid nitrogen storage, and 100 μL of the medium was added to the top of the frozen cells in each cryotube before the vials were placed into a 37°C water bath in order to shorten the thawing time. The cells were quickly thawed after swirling the cryovials in the 37°C water bath for a maximum of 1 min and then transferred to 15-mL tubes where a total of 12 mL medium was added with spaced time intervals for a gentle thawing process. The cells were then centrifuged in 300 × *g* for 10 min and washed a total of three times with RPMI medium. The cells were at last resuspended in 2 -mL medium. Cell concentration and viability were assessed by Solution 13 and counted using the NC-3000.

### Separation of CD4^+^ CD25^+^ Tregs

To enrich the Treg population, Treg isolation kits separating CD4^+^ CD25^+^ T cells (Miltenyi Biotech Norden AB, Lund, Sweden) were used. Cell separation was performed following the manufacturer’s description. CD4^+^ T cells were negatively separated using an LD column (Miltenyi Biotech). The negatively selected CD4^+^ cells were then positively selected through the use of CD25 MicroBeads (Miltenyi Biotech, 10 µL per 10^7^ cell) and an MS Column (Miltenyi Biotech). Approximately 10^6^ cells were collected from 130 × 10^6^ PBMCs. The cells were stained for CD25 and CD127 where approximately 75% were of the CD4^+^ CD25^+^ CD127^low^ phenotype.

### Cell proliferation assays

Responder PBMCs (n = 5) were then stained with CellTrace Violet Cell Proliferation Kit (ThermoFisher Scientific) according to the company’s protocol. In this process, the stock of CellTrace Violet staining (ThermoFisher Scientific) was diluted 2000 times with 5 -mL PBS. To stop the reaction, TexMACS Medium (Miltenyi Biotech) was added. 2 × 10^5^/well CellTrace-labeled responder PBMCs were then aliquoted into the 96 U-bottomed microculture plates. Triplicates were made in each group. The labeled responder cells with (1) culture with medium alone (negative control), (2) culture with anti-CD3/CD28 antibodies (positive control, Mabtech AB, Nacka, Sweden), culture with anti-CD3/CD28 antibodies plus CD4^+^ CD25^+^ Treg in varying ratios: (3) responder and Treg ratio of 1:1, (4) 1:0.5, and (5) 1:0.25. The cells were then incubated at 37°C with 5% CO_2_ humidified air for 5 days. Cells were then harvested for analysis with flow cytometry.

### Flow cytometry

1 × 10^6^ cells for lineage staining and 2 × 10^6^ cells for Treg staining were aliquoted to fluorescence-activated cell sorting (FACS) tubes. The cells were then diluted with PBS to 2 mL and washed (centrifuged at 350 × *g* for 5 min). To identify dead cells, FVS 520 viability stain (BD Horizon, NJ, USA) was added to cells stained with the lineage panel, and FVS 510 viability stain (BD Horizon) was used for the Treg panel. The cells were then washed with FCS buffer (PBS with 1% FBS) and resuspended in 100-µL buffer. Surface staining antibodies were prepared for both lineage and Treg staining. The antibodies (Table 1) were added to each cell suspension, and the cells were incubated for 15 min in the dark. Cells were washed once, and lineage-stained cells were then resuspended with 300-μL buffer.

**Table 1.** Fluorophore, clone, and manufacturer for each respective antibody used in the experiments.

Antibody	Fluorophore	Clone	Manufacturer
CD3	V450	UCHT1	BD Horizon
CD4	PerCP-Cy5.5	L200	BD Pharmingen
CD8	APC-H7	SK1	BD Pharmingen
CD14	PE-Cy7	M5E2	BD Biosciences
CD19	V500	HIB19	BD Horizon
CD45	APC	HI30	BD Pharmingen
CD16	PE	3G8	BD Pharmingen
CD56	PE	B159	BD Pharmingen
CD25	PE-Cy7	BC96	BioLegend
CD127	PE	hIL-7R-M21	BD Pharmingen
FoxP3	AF647	236A/E7	BD Pharmingen

For Treg staining, after cell surface staining (see above), cells were fixed and permeabilized by using a Fixation/Permeabilization concentrate (Invitrogen, MA, USA) diluted 1:4 with Fixation/Perm Diluent (Invitrogen) solutions. After incubation at 4°C for 40 min, the cells were washed two times with Permeabilization buffer (Invitrogen), and the anti-FoxP3 antibody or isotype control antibody was subsequently added. After 40 min of incubation at 4°C, the cells were washed two times with the Permeabilization buffer and then resuspended with 350-μL buffer. Cell samples were then acquired on the flow cytometer (BD FACS Canto RUO Special Order System, BD Biosciences). The results were analyzed using FlowJo v10.8 (BD Biosciences).

#### Gating strategy—Lineage panel

**Figure 1.**
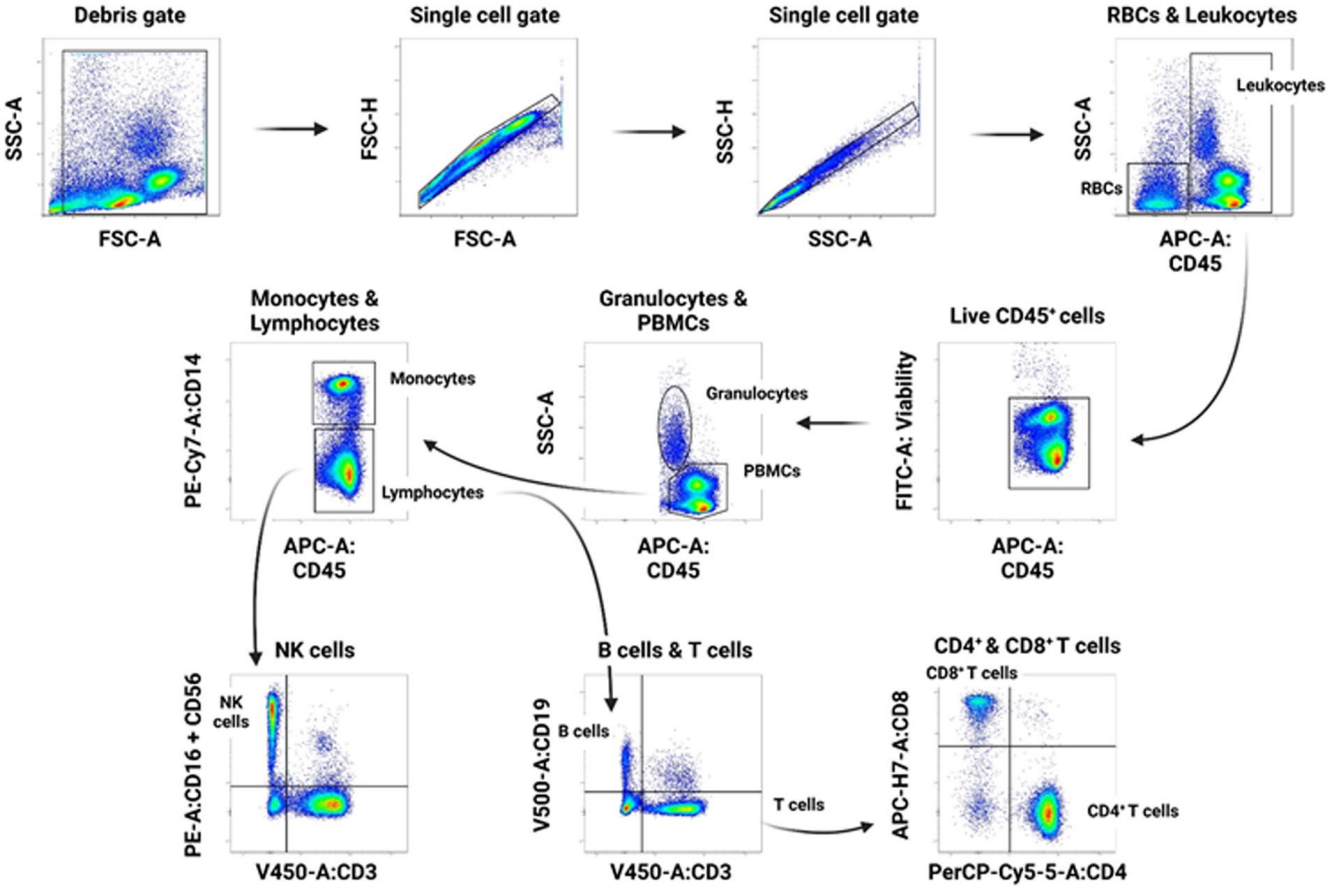
Gating strategy for the lineage panel. Single cells were gated out of the debris gate, and then leukocytes and RBCs were separated in the SSC-A vs CD45 gate. CD45- cells were RBCs and CD45^+^ were leukocytes which were then gated for viability. Granulocytes and PBMCs were separated in SSC-A vs CD45 gate where the more granular granulocytes were SSC-A high, CD45^+^ and PBMCs SSC-A intermediate or low, CD45^++^. PBMCs were then gated for monocytes (CD14^+^ CD45^+^) and lymphocytes (CD14^−^ CD45^+^). Lymphocytes were separated for NK cells (CD3^−^ CD16^+^ CD56^+^) and B cells (CD3^−^ CD19^+^). CD3^+^ cells were at last gated for CD4^+^ and CD8^+^ T cells. PBMCs: Peripheral blood mononuclear cells. SSC-A: Side scatter area. FSC-A: Forward scatter area.

As illustrated in Fig. 1, blood gating was done in side scatter area (SSC-A) vs forward scatter area (FSC-A) to exclude debris according to granularity and size, and then single cells were gated. Leukocytes and RBCs were separated in the SSC-A vs CD45 gate. We further confirmed that RBC-gated cells (CD45 negative and low SSC-A) were all smaller than lymphocytes. Thereafter, CD45^+^ cells were gated for viability; the live CD45^+^ were then gated for the more granular granulocytes (SSC-A high, CD45^+^) and PBMCs (SSC-A intermediate or low, CD45^++^). PBMCs were further gated for monocytes (CD14^+^ CD45^+^) and lymphocytes (CD14^−^ CD45^+^). Lymphocytes were further separated for CD3^−^ CD16^+^ CD56^+^ natural killer (NK) cells, CD3^−^ CD19^+^ B cells, and CD3^+^ CD19^−^ T cells. CD3^+^ T cells were then gated further into CD8^+^ vs CD4^+^ T cells.

#### Gating strategy—Treg panel

**Figure 2.**
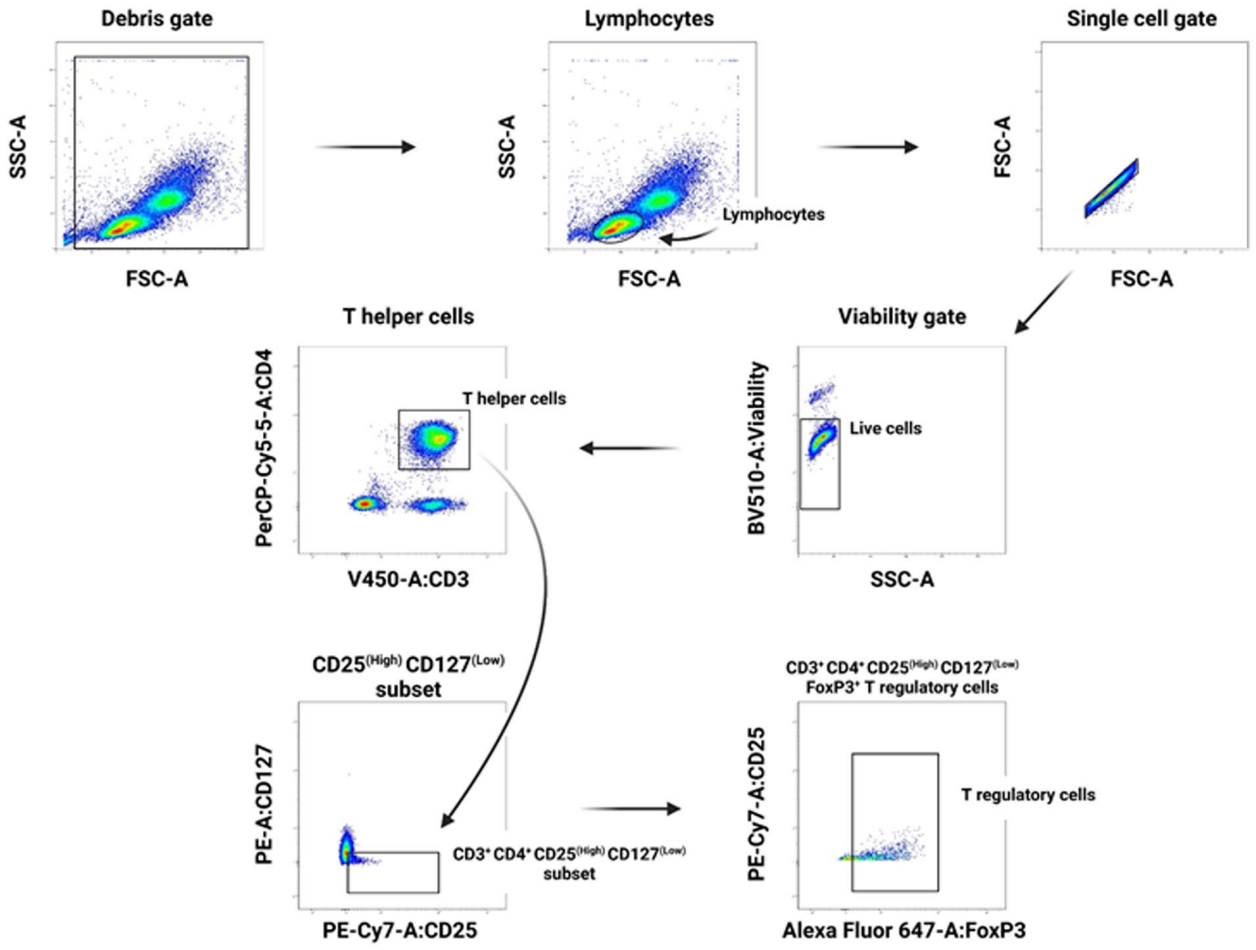
Gating strategy for T regulatory cell panel. Lymphocytes were gated out of SSC-A vs FSC-A gate based on morphology. After single cell and live cell gating, T-helper cells (CD3^+^ CD4^+^) were gated out. CD3^+^ CD4^+^ T-helper cells were then gated for the CD25^High^ CD127^Low^ subset where non-stained cells were used as a baseline for comparisons in the gating process of this. The latter subset was lastly gated for CD3^+^ CD4^+^ CD25^High^ CD127^Low^ FoxP3^+^ Treg cells. CD25 vs FoxP3 gating was determined by FoxP3 isotype where FoxP3^−^ was identified as a reference (gating not shown).

Illustrated in Fig. 2, after gating for single live cells, CD3^+^ CD4^+^ were gated, out of which the CD25^+^ and CD127^Low^ were identified and gated where non-stained cells were used as a baseline for comparisons in making the gating. Lastly, the FoxP3^+^ subset was identified with FoxP3^+^ isotype gating as a reference (gating not shown) to isolate CD4^+^ CD25^+^ CD127^Low^ FoxP3^+^ Treg cells.

#### Gating strategy—Cell proliferation

**Figure 3.**
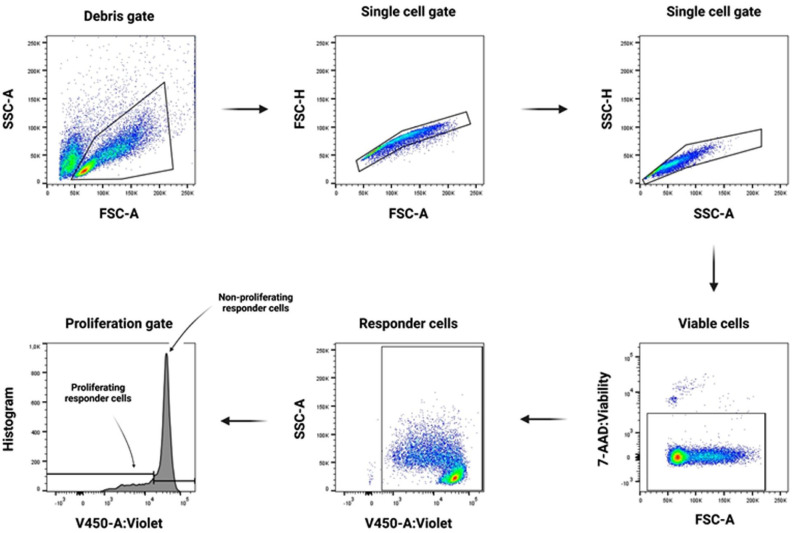
Gating strategy for cell proliferation assays. Lymphocytes and proliferating cells were gated in the SSC-A vs FSC-A plot with enough margins not to miss out on potential proliferating cells. After single cell gating, live cells were determined in 7-AADD: viability vs FSC-A gate. Live cells were gated in SSC-A vs V450-A: Violet plot to separate out potential Treg cells (SSC-A^Low^ V450-A: Violet^−^). Lastly, ~98% of V450-A: Violet^++^ cells were deemed to be non-proliferating, and the rest V450-A: Violet^Intermediate/Low^ was consisting of proliferating responder cells.

Fig. 3 demonstrates how viable cells were gated according to 7AAD-A negative vs FSC-A. The cell proliferation was gated through a histogram where a threshold of around 98% of cells was deemed violet positive (non-proliferating), and the rest violet negative (proliferating).

### Cell culture medium

Medium for ELISpot assay, RPMI-1640 (containing GlutaMAX and Hepes), was supplemented with 5% FBS and penicillin 100 U/mL and streptomycin 100 µg/mL (ThermoFisher Scientific). For the proliferation assay, a TexMACS medium (Miltenyi Biotech) supplemented with penicillin and streptomycin was used.

### Enzyme-linked immunosorbent spot (ELISpot) assay

ELISpot plate assays were performed according to the manufacturer’s protocol for both interferon gamma (IFN-γ) and ApoE/IL-6 plates (Mabtech AB). The capture antibody pre-coated plates were pre-wet with 100 µL of culture medium for > 30 min at room temperature. The medium was discarded from the ELISpot plates just before responder PBMCs were added. As responder cells, 2 × 10^6^/mL PBMCs in culture medium were prepared, and 100 µL/well cell suspension was added to each well.

IFN-γ ELISpot assays were used to detect memory T-cell activities against common viral peptides. The stimuli were prepared in culture medium with peptides (2 µg/mL, CEF, CEFTA & CMV, Mabtech AB) and anti-CD3/CD28 antibodies (100 ng/mL, clone CD3-2, CD28-A, Mabtech AB). ApoE/IL-6 ELISpot assays were used to examine monocyte function. Transforming growth factor-β (TGF-β) (10 ng/mL, PeproTech, London, UK) and lipopolysaccharide (LPS) (055/B5, 10 ng/mL, Merck KGaA, Darmstadt, Germany) were added to the wells as stimuli.

Subsequently, 100 µL/well of each stimulus was added to the respective wells. The plates were incubated at 37°C with 5% CO_2_ in a humidified atmosphere for 40 h. Cytokine-producing cell-detection procedure was performed according to manufacturer’s recommendations. In brief, after washing plates to remove cultured cells, the plates were incubated with 100 µL/well of biotinylated detection antibody, 1 µg/mL in PBS with 0.2% bovine serum albumin (BSA) for 1 h. Non-bound antibodies were washed away, followed by 1-h incubation with 100 µL/well of alkaline phosphatase-conjugated Streptavidin (SA-ALP, Mabtech AB). To visualize spots, 100 µL of BCIP/NBT substrate (Mabtech AB) was used per well. The reaction was stopped by washing the plates with tap water, and the plates were dried until analysis. The spot number was analyzed by IRIS (Mabtech AB).

### RNA preparation

RNA preparation was done with PureLink RNA Mini Kit (ThermoFisher Scientific) according to the enclosed protocol. RNA was prepared from 5 × 10^6^ PBMCs. RNA content was measured by using a spectrophotometer (LabVision Nano, LabVision AB, Värmdö, Sweden). 600 ng of RNA was converted to cDNA by using a cDNA synthesis kit (ThermoFisher Scientific). Quantification of mRNA was performed using TaqMan real-time PCR on an Applied Biosystems 7500 Fast Real-Time PCR System (ThermoFisher Scientific). The relative gene expressions of IL-6, IL-1β, TNF-α, IL-10, IFN-γ, FoxP3, and CD25 were examined, and all samples were analyzed in triplicate. Relative mRNA expression was calculated from the Ct-values against the housekeeping genes peptidyl-prolyl cis-trans isomerase and glyceraldehyde-3-phosphate dehydrogenase (Table 2).

**Table 2.** TaqMan primers/probes targeting specific mRNAs used for mRNA quantification.

Gene	ID
Peptidyl-prolyl cis-trans isomerase A (PPIA)	Hs99999901_g1
Glyceraldehyde-3-phosphate dehydrogenase (GAPDH)	Hs02786624_g1
Human TNF-α	Hs00176128_m1
Human IL-1β	Hs01555410_m1
Human IL-6	Hs00174131_m1
Human Foxp3	Hs01085834_m1
Human ISG20 (CD25)	Hs00158122_m1
Human IFN-γ	Hs00980290_g1
Human IL-10	Hs00961622_m1

mRNA: messenger ribonucleic acid, TNF: tumor necrosis factor, IL: interleukin, CD: cluster of differentiation, IFN: interferon.

### Statistical analysis

A comparison of two groups was performed by the Wilcoxon test. A linear regression analysis was performed for proliferation assays. GraphPad Prism version 9.5.0. (Dotmatics, MA, USA) was used for the statistical tests. Results in the text are shown as the geometric mean and geometric standard deviation (SD).

## Results

### ACK treatment did not change leukocyte composition but increased memory T-cell activity against virus peptides

**Figure 4.**
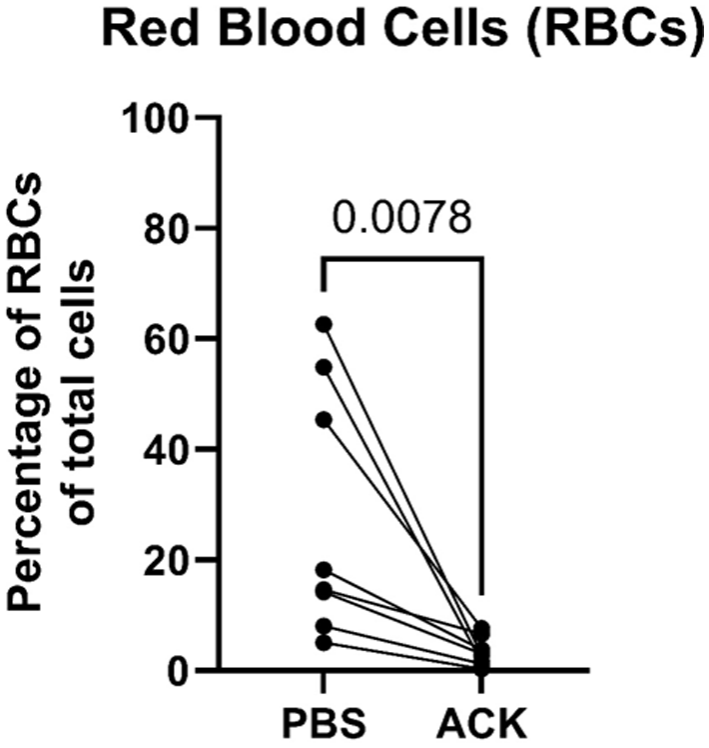
Wilcoxon test performed to determine red blood cell (RBC) proportion out of all cells in ACK-treated and PBS-control groups. ACK: ammonium-chloride-potassium; PBS: phosphate-buffered saline.

PBMCs purified with gradient centrifugation are often contaminated with a small portion of RBCs and granulocytes, which was inevitable in our case as well. ACK treatment removed the contaminated RBCs efficiently (Fig. 4, mean ± SD, 27.9 ± 22.7% vs 3.4 ± 2.6%, PBS vs ACK, *p* = 0.0078). Leukocyte viability was not affected by ACK treatment, which was examined by the NucleoCounter NC-3000 as well as flow cytometry using FVS dyes (mean ± SD, 98.7 ± 1.0% vs 97.3 ± 2.5% for PBS vs ACK, respectively, in flow cytometry analysis). ACK treatment did not change the cell composition for monocytes and lymphocyte subsets of NK cells, T cells, and T-cell subsets (CD4^+^ T cells, CD8^+^ T cells, and CD3^+^ CD4^+^ CD25^+^ CD127^Low^ FoxP3^+^ Treg cells). The summary of cell composition is shown in Table 3.

**Table 3.** Comparison of the geometric mean of cell proportions of freshly enriched PBMCs between the ACK-treated group vs the PBS-incubated group.

Mean ± SD	ACK	PBS	*p*-value
Leukocytes^a^	96.13 ± 2.762	68.89 ± 25.24	0.0078*
Live CD45^+^ cells^b^	98.19 ± 1.706	98.35 ± 1.403	0.9453
RBCs^a^	3.371 ± 2.596	27.87 ± 22.72	0.0078*
PBMCs^c^	89.64 ± 14.18	83.38 ± 18.42	0.0156*
Granulocytes^c^	8.23 ± 12.59	13.52 ± 16.54	0.0234*
Lymphocytes^d^	87.53 ± 9.410	86.03 ± 11.35	0.4375
Monocytes^d^	12.36 ± 9.383	13.84 ± 11.27	0.4609
NK cells^e^	13.61 ± 9.416	14.13 ± 9.294	0.25
B cells^e^	6.753 ± 4.905	5.748 ± 4.460	0.0234*
T cells^e^	75.7 ± 10.61	75.51 ± 10.93	0.8125
CD4^+^ T cells^f^	64.34 ± 13.10	66.31 ± 12.12	0.7422
Treg cells^g^	3.89 ± 2.576	4.263 ± 2.611	0.2188
CD8^+^ T cells^f^	31.01 ± 12.40	29.05 ± 11.43	0.6406

Note that contamination of red blood cells (RBCs) and granulocytes still exists after enrichment of peripheral blood mononuclear cells (PBMCs). Please refer to the cell separation section of material and methods for the reasoning. The mean and standard deviation (SD) with corresponding *p*-values are shown (n = 8). ACK: ammonium-chloride-potassium; PBS: phosphate-buffered saline.

a % of all single cells (see Fig. 1).

b % of leukocyte gate.

c % in live CD45^+^ cells.

d % in PBMC gate.

e % in lymphocyte gate.

f % in CD3^+^ T-cell gate.

g % in CD4^+^ T-cell gate.

* Statistically significant result in the Wilcoxon analysis.

RNA was extracted from cell samples treated with or without ACK, and the relative expression of various cytokines was compared. There were no significant differences in the relative gene expression between ACK-treated and control samples for the analyzed cytokines or Treg-related genes (IL-6, IL-1β, TNF-α, IL-10, IFN-γ, FoxP3, and CD25) (data not shown).

**Figure 5.**
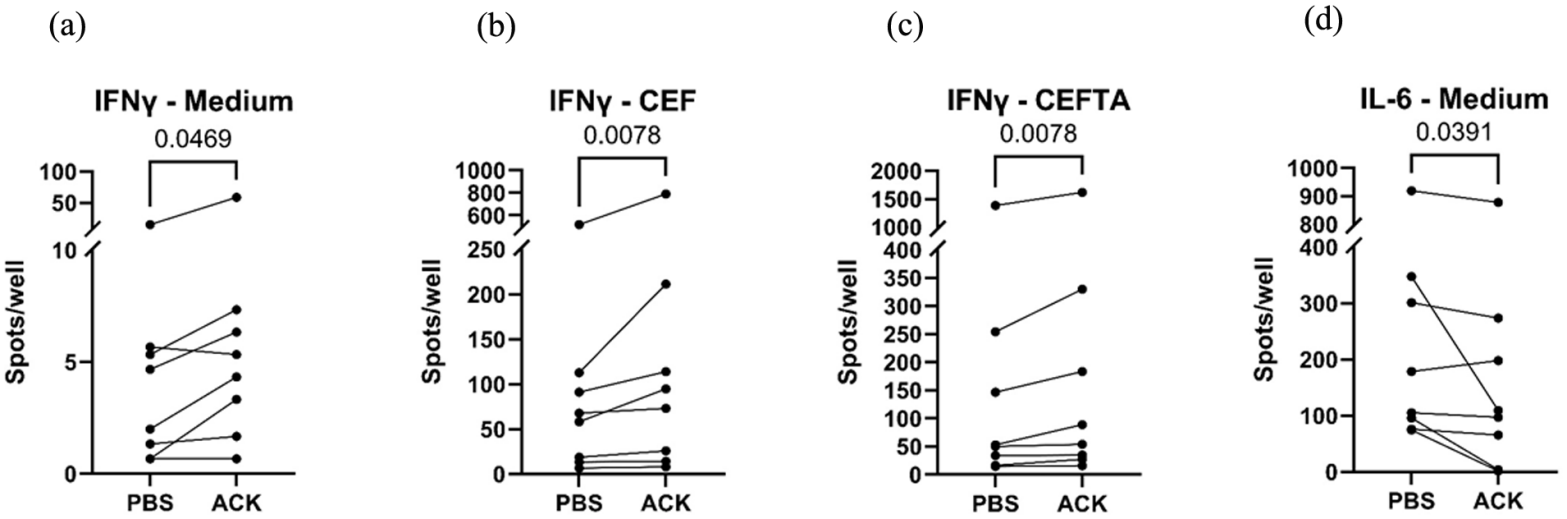
Wilcoxon test to determine cytokine producing cell spot counts. IFN-γ-producing cell spot count in ACK vs PBS groups in (a) medium, (b) CEF stimulation, and (c) CEFTA stimulation. (d) IL-6-producing cell spot count in ACK vs PBS groups in medium. ACK: ammonium-chloride-potassium; IFN-γ: interferon gamma; PBS: phosphate-buffered saline.

The memory T-cell function was analyzed by IFN-γ ELISpot after being activated with CEF/CEFTA virus peptides. The spontaneous IFN-γ-producing cell number was generally low. However, we noted that in the ACK-treated group, spontaneous IFN-γ-producing cell numbers were significantly higher than those of PBS-control groups, even in medium alone (mean ± SD, 4.4 ± 4.6 vs 11 ± 19.3, *p* = 0.0469, PBS vs ACK, respectively) (Fig. 5a). Memory T-cell activities against virus peptides were also stronger in the ACK-treated group than in the PBS-control group, that is, with CEF stimulation (mean ± SD, 111 ± 169 vs 166 ± 261, PBS vs ACK, *p* = 0.0078) and CEFTA stimulation (mean ± SD, 244 ± 469 vs 294 ± 545, PBS vs ACK, *p* = 0.0078) (Fig. 5b, c). It is also noted that ACK-treated cells showed a decrease in spontaneous production of IL-6 compared to PBS-control samples (Fig. 5d, mean ± SD, 262 ± 285 vs 203 ± 288, PBS vs ACK, *p* = 0.0391). ApoE production did not significantly differ with or without ACK treatment (data not shown).

### Cryopreservation reduced RBC, granulocyte, and T-helper cell populations, but Treg function remained intact

**Figure 6.**
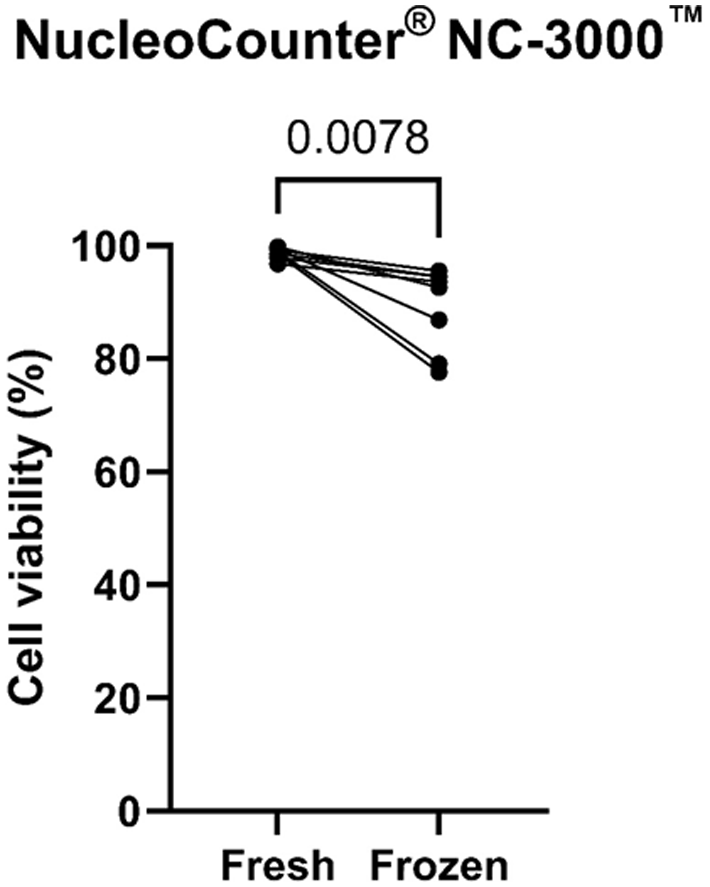
Cell viability between fresh and cryopreserved enriched peripheral blood mononuclear cells measured in NucleoCounter NC-3000 (Chemometec, Lillerød, Denmark). Statistics done with Wilcoxon test.

**Figure 7.**
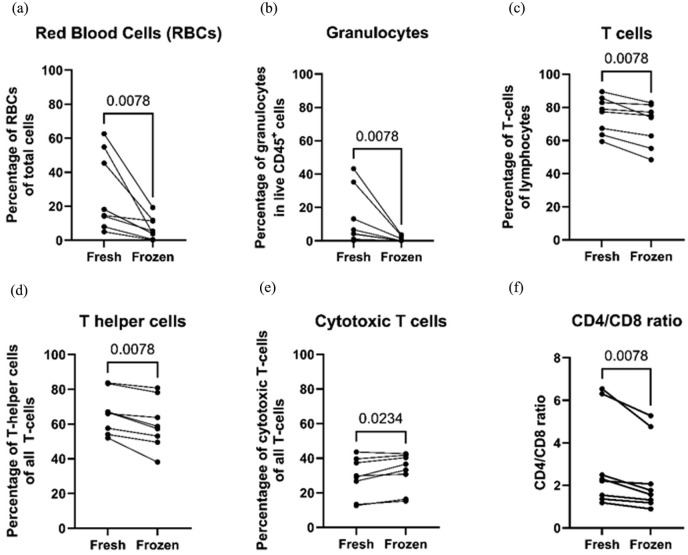
Comparison between fresh and cryopreserved enriched peripheral blood mononuclear cell samples regarding cell composition. (a) red blood cells (RBCs), (b) granulocytes, (c) T cells, (d) T helper cells, (e) cytotoxic T cells, and (f) CD4/CD8 T-cell ratio. Wilcoxon test was used for all comparisons.

Cryopreservation with 10% DMSO led to a significant decrease in cell viability, confirmed by viability staining and counting (Fig. 6) and by flow cytometry (data not shown). Both RBCs and granulocytes showed a substantial decrease after cryopreservation (Fig. 7a, b for RBCs and granulocytes, respectively). To a lesser extent, the proportion of CD3^+^ T cells in the lymphocyte gate and CD4^+^ T cells also decreased after cryopreservation (Fig. 7c, d for CD3^+^ T cells and CD4^+^ T cells, respectively). On the contrary, NK cells and CD8^+^ T-cell proportions increased among PBMCs (Fig. 7e for CD8^+^ T cells, graph not shown for NK cells). The CD4^+^/CD8^+^ T-cell ratio decreased in frozen cell samples (Fig. 7f). There was no observable difference for lymphocytes, monocytes, and B cells (data not shown). The proportion of CD3^+^ CD4^+^ CD25^+^ CD127^Low^ FoxP3^+^ Treg in the CD4^+^ T-helper cell population did not change between fresh and cryopreserved cells (mean ± SD, 4.263 ± 2.611 vs 2.941 ± 1.972 for fresh vs frozen, respectively, *p* = 0.3750). The summary of the composition analysis comparing fresh vs frozen PBMCs is shown in the Supplemental Material.

**Figure 8.**
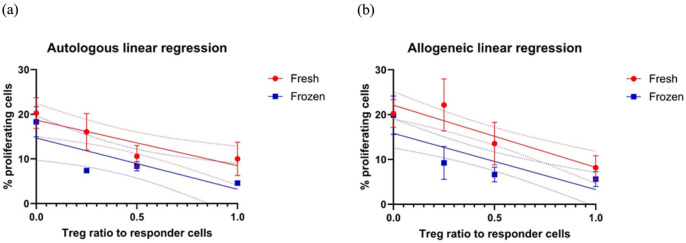
Linear regression models to compare percentage of proliferating responder cells (y-axis) with regulatory T cell ratio with responder cells (Treg: Tresp, x-axis) (n = 5). Red lines are fresh cells, and blue lines are cryopreserved cells. 95% interval of the best-fit line outlined as dotted lines. (a) Autologous co-cultures (R^2^ 0,6363) and (b) allogeneic co-cultures (R^2^ 0,5448) with regulatory T cells and responder cells.

Cell proliferation assays showed that responder T cells were able to be stimulated and proliferate equally well both in fresh and frozen samples (data not shown). In linear regression models, there was no statistical difference in suppressive activity between fresh and frozen Tregs in autologous and allogeneic co-cultures (R^2^ 0,6363 and 0,5448, respectively, Fig. 8a, b).

Cryopreserved PBMCs exhibited a significantly higher number of IFN-γ-producing cells when stimulated by CEF peptides than fresh PBMCs (mean ± SD, 111 ± 169 vs. 581 ± 878 fresh vs. frozen cells, respectively, *p* = 0.0391). The responses against other virus peptides were similar between fresh and cryopreserved PBMC.

**Figure 9.**
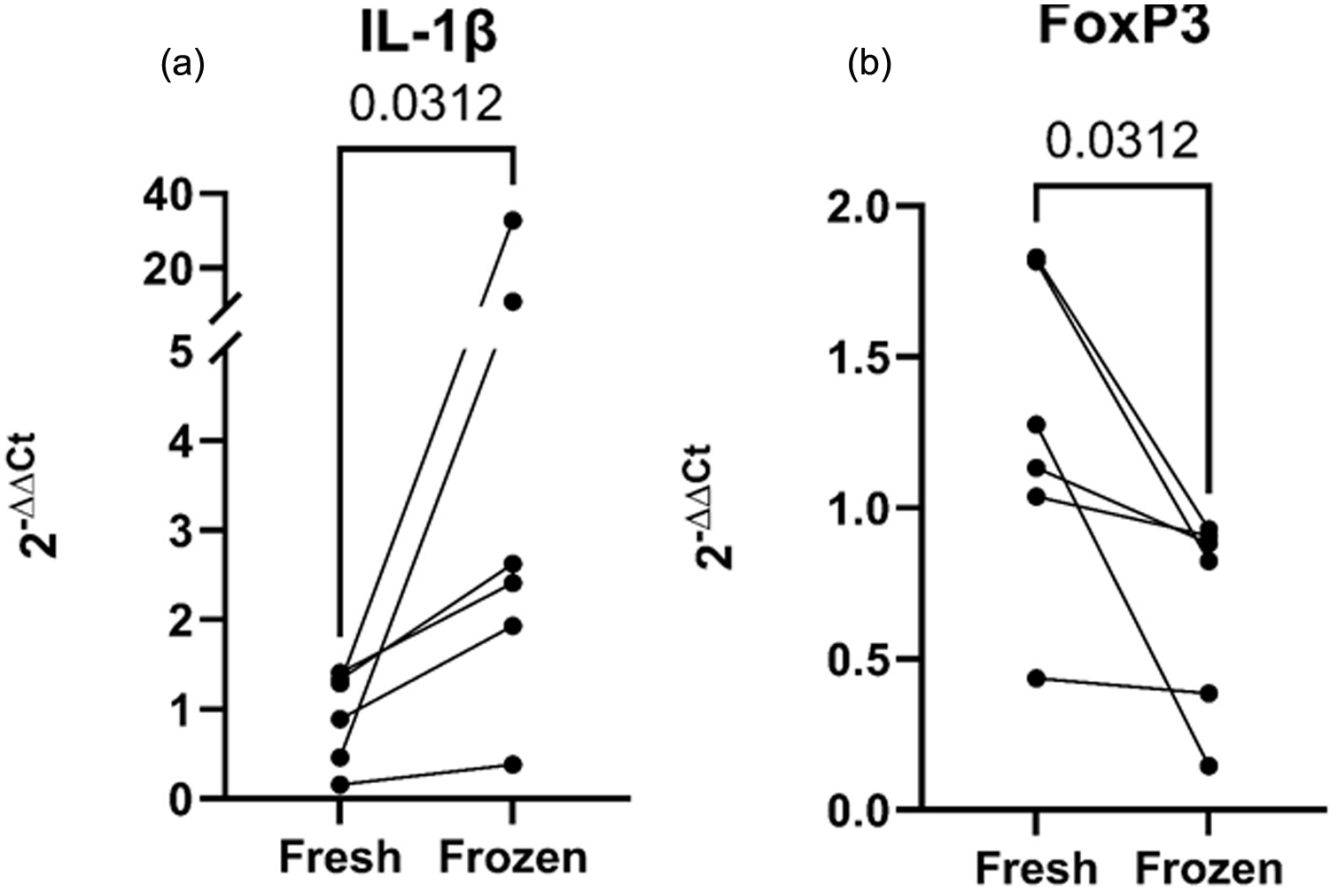
Relative gene expression measured through quantitative polymerase chain reaction (qPCR). Y-axis denotes the fold change, and the x-axis represents fresh or cryopreserved (frozen) cells. Relative gene expression of (a) IL-1β and (b) FoxP3 is illustrated as significant statistical findings through the Wilcoxon test.

Relative gene expression analysis showed an increase in IL-1β expression, while FoxP3 expression was lower in cryopreserved cells than in fresh cells (Fig. 9a, b).

## Discussion

In this study, we evaluated the effects of RBC lysing buffer (ACK) as well as short-time cryopreservation on PBMC phenotype, functionality, and relative gene expression of both pro- and anti-inflammatory genes in PBMC-enriched cells. Moreover, we evaluated the effects of cryopreservation on Tregs and elucidated their suppressive abilities post-thaw on proliferating cells.

ACK treatment efficiently reduced RBCs and did not affect leukocyte viability. The composition of the PBMCs remained unchanged with ACK treatment, including T helper cells and Tregs. Cells with ACK treatment exhibited stronger memory T-cell responses against different viral peptides, as shown by the IFN-γ ELISpot assays. ACK-treated cells also showed a reduced IL-6 selection compared to non-ACK-treated cells. Proinflammatory cytokine gene expression and Treg-related gene expressions did not show significant differences with or without ACK treatment.

The RBC lysing effect of ACK is through an accumulation of ammonium chloride within the cell, increasing the pressure in the cell, which results in a rupture of the RBC membrane^13^. Bossuyt et al.^13^ pointed out that ACK treatment produced a substantial amount of debris and may change some antigen presentation intensity (i.e., lowered CD4-staining intensity) and thereby change the CD4/CD8 ratio which was not observed in our experiments. We used enriched PBMCs contaminated with RBCs and granulocytes to varying degrees where three specific buffy coats had an overrepresentation of RBCs and granulocytes that could indicate a suboptimal PBMC isolation in those samples.

A comparative study examining RBC lysing buffers looked at a lysis buffer containing NH_4_Cl called IOTest 3 Solution (Immunotech SAS, Beckman Coulter, CA, USA) that was deemed very efficient in RBC lysis (median leukocyte count 96.85%), and where there were no major leukocyte subset deviations in cell percentages apart from a slight relative increase in CD14^+^ monocytes^14^. In our study, we observed that ACK treatment did not significantly affect the compositions of PMBC populations, including Treg populations. Unlike the two studies mentioned earlier, which used whole blood for analysis, we used gradient-enriched PBMCs. Even though there were impurities in the separation of PBMCs, the amount of contaminated RBCs and granulocytes was significantly less than that in whole blood. Therefore, the amount of debris after RBC lysis in our system could be much lower than that when using whole peripheral blood, which may affect the cell activities and composition differences.

Spontaneous release of IL-6 was reduced in ACK-treated cells compared to non-ACK-treated cells, though we did not find any differences in proinflammatory gene expression with or without ACK treatment. We also noted that ACK-treated responder cells released lower levels of proinflammatory cytokines than non-ACK-treated responder cells^15^. This finding may suggest that ACK could potentially change membrane permeability and stimulate the release of granules in the cytoplasm without increasing mRNA. Further studies are needed to understand the mechanisms.

The effect of ACK treatment on antigen-presenting cells and memory T-cell functions has not been well studied. Here, we used ELISpot to investigate memory T-cell function against virus peptides. ACK-treated cells exhibited higher IFN-γ production than non-ACK-treated cells in both medium alone and stimulation with CEF and CEFTA peptides, suggesting that ACK treatment of freshly separated cells increased the sensitivity of both CD4^+^ and CD8^+^ T cells against virus peptides. Previous ELISpot assays have shown that granulocyte contamination greatly affects the spot quality and quantity^16^, which indicates that contaminated granulocytes may interact with memory T-cell activities against virus peptides in *in vitro* culture. Therefore, a reduction in granulocytes, as we saw after ACK treatment, could explain the increase in IFN-γ production. The latter effect may be beneficial in a clinical context where ACK treatment could increase the functionality of T cells.

The effects of cryopreservation of PBMCs were also evaluated in this study. Cell viability was significantly reduced after cryopreservation. Furthermore, cryopreserved cell samples revealed substantial decreases in the proportion of RBCs and granulocytes. Interestingly, the three cell samples with the highest proportion of RBCs and granulocytes also exhibited the lowest cell viability after cryopreservation, and the reason for this could be the susceptibility of RBCs and granulocytes to cryopreservation, thus lowering the overall viability. CD14^+^ monocyte population and the proportion of CD19^+^ B cells in lymphocytes did not change, but CD3^+^ T cells in the lymphocyte gate and CD4^+^ T cells in the T-cell population decreased after cryopreservation. A slight but significant increase of NK cells and CD8^+^ T-cell populations in lymphocytes was noted after cryopreservation. CD4^+^/CD8^+^ T-cell ratio showed a decrease after cryopreservation, which is attributed to the decreased proportions of CD4^+^ T cells and the increase in CD8^+^ T cells. There were no changes in Treg populations after cryopreservation. The functionality of PBMCs was generally stable with cryopreservation. Fresh and frozen Treg did not differ statistically in their suppressive abilities after cryopreservation. The relative gene expression revealed an increase in IL-1β expression but a decrease in FoxP3 in frozen cells compared to those in fresh cells.

It is known that freeze-thaw cycles reduce cell yield and viability^17,18^, which we observed in our study as well. It is worth noting that the substantial loss of cells was primarily observed after post-thaw washing (mainly to remove DMSO), rather than immediately after thawing. Flow cytometry of these thawed and washed cells revealed that RBC and granulocyte population were significantly reduced. Post-thaw washing is necessary *in vitro* to wash out DMSO, which can affect cellular functionality, and there are therefore several different aspects that can affect cell viability after thawing, that is, cryopreservation itself, post-thaw washing, and/or DMSO toxicity. Regarding granulocytes, Boonlayangoor et al.^19^ reported that after cryopreservation with 10% DMSO, the phagocytic ability and bactericidal effects decreased, and morphological alterations with nuclear swelling were observed, indicating cellular damage to the granulocytes. Another study has also shown a similar morphological picture of granulocytes post-cryopreservation using electron microscopy and that the reduced granulocyte functionality could, to some degree, be alleviated if the DMSO dilution occurred at 37°C compared to 4°C^20^.

It has previously been reported that the monocyte population decreases significantly after cryopreservation^18,21^. In our study, PBMCs were cryopreserved from 1 week up to 1 month, and we did not observe any difference between the frozen monocyte population and the population in fresh cell samples. Another study looking at B cells pointed out that the cell population remained stable during the first 2 months of cryopreservation at −80°C but gradually decreased after cryopreservation over a 12-month period^22^. B cell composition remained intact in our study.

The effect of cryopreservation on T-cell composition has been studied by many groups. We noted a slight but statistically significant decrease in the CD4^+^ T cell percentage and a corresponding CD8^+^ T-cell increase after cryopreservation compared to fresh cells. We did not find any difference in Treg populations between fresh and frozen cells. Previous studies have had disparities in the results where one study has shown that the percentage of CD4^+^ and CD8^+^ T cells were unchanged after cryopreservation^[Bibr bibr22-09636897251382315] 23^, while another found that cryopreservation had no effect on the expression of immune markers of innate and adaptive immune cells^24^. One study found the CD8^+^ T-cell proportions to be significantly reduced^18^ which runs contrary to our findings.

In terms of cryopreservation of Treg, one group found that the population was in general stable after cryopreservation in terms of cell percentages^25^ while another showed that cryopreservation decreased the proportion of Treg^26^. Gołąb et al.^27^ came to a similar finding as the latter but also noticed an increase in Treg proportions after restimulation and concluded that Tregs can benefit from *ex vivo* restimulation after thawing.

Previous experiments have shown a decreased suppressive activity of Tregs after cryopreservation, which could be restored by post-thaw expansion and where a dose-dependent suppressive effect was present^28^. A study using PBMCs from baboons also showed that cryopreservation with 10% DMSO significantly decreased the suppressive function of Tregs, but that the function was restored, and to a degree even better after post-thaw reactivation^29^. Likewise, another study using a similar experimental setup with PBMCs from cynomolgus monkeys found that *ex vivo* expanded Tregs maintained phenotypic characteristics but had impaired viability, which could be restored after post-thaw stimulation/expansion. The same study also showed that cryopreserved *ex vivo* expanded Tregs maintained their suppressive functionality in both allogeneic and autologous responder cell co-cultures in a dose-dependent manner^30^. Our study did not indicate any difference in Treg function between fresh and frozen cell samples in linear regression models, although the sample number for proliferation assays was low (n = 5), and we still see this as a field where more research is needed in order to elucidate these effects.

Recently, in our department at Karolinska Institutet, Stockholm, Sweden, in collaboration with Professor Korsgren’s team (Rudbeck Laboratory, Uppsala University, Uppsala, Sweden), co-injection of enriched autologous Treg components and pancreatic islet transplantations were performed with five patients, which exhibited full safety, with no sign of acceleration of allograft rejection^31^. In this study, an autologous Treg-enriched component was prepared and then cryopreserved in a controlled manner. A significant reduction in cell yield was reported, but the functionality of thawed cells was not evaluated. Furthermore, the treatment efficacy was not fully evaluated because the injected cell number varied, and immunosuppressive treatment was not adjusted for cell therapy (patients had induction therapy with Basiliximab and maintained on Tacrolimus/Everolimus).

Regarding PBMC functionality after cryopreservation, it has been reported that CD4^+^ and CD8^+^ T cells were not affected in the production of IFN-γ, IL-2, IL-4, and IL-5 when stimulated with Epstein-Barr virus (EBV), Cytomegalovirus (CMV), mumps, grass, and dust antigens in ELISpots assay^32^. A previous study reported that, in general, cryopreserved cells produced higher amounts of cytokines after stimulation with different Toll-like receptor (TLR) ligands, though no differences were present for IFN-γ^24^. This can, to some degree, provide insight into how PBMCs are affected functionally by cryopreservation. Cell function could theoretically recover by letting the PBMCs rest overnight in order to decrease the burden of apoptotic cells, and Santos et al.^33^ showed using ELISpot assays that this was greatly beneficial for increasing the cytokine response after cryopreservation. Although such a protocol was not implemented in our study, we did find an increase in memory cell functions against virus peptides in the IFN-γ ELISpot assays. Our results furthermore showed that frozen PBMCs produced higher levels of IFN-γ under CEF stimulation than fresh PBMCs, which could partly be attributed to the increased proportion of CD8^+^ T cells.

In comparing relative gene expression, we found that IL-1β increased in cryopreserved samples while FoxP3 decreased. Yang et al.^34^ used microarray gene expression analysis and found 1327 genes whose expression was changed more than three-fold after cryopreservation where a little more than half of the analyzed amount increased, and the remaining genes decreased in expression indicating that cryopreservation has a general effect on gene expression. Moreover, DMSO has been reported to enhance mRNA expression of IL-1β in conjunction with LPS induction, although DMSO alone did not increase IL-1β mRNA expression, indicating that other cellular stimulations are needed furthermore^35^. It has also been reported that inflammatory conditions, including IL-6, induce a decrease in FoxP3 gene expression through epigenetic methylation of CpG DNA^36^. Kivling et al.^37^ did however not find any difference in the spontaneous relative gene expression of the Treg-associated markers CTLA-4, sCTLA-4, TGF-β, and FoxP3 after cryopreservation. FoxP3 mRNA expression did, however, decrease when thawed cryopreserved cells were stimulated with phytohemagglutinin (PHA), tetanus toxoid (TT), and β-lactoglobulin (βLG), and the authors discussed that cryopreservation could be suitable to detect Treg markers if cell handling is performed carefully and without antigen stimulation^37^.

Our results indicate that the Treg population within the CD4^+^ T-cell population remained unchanged; however, the CD4^+^ T-cell population decreased after cryopreservation, resulting in a corresponding decrease in the Treg population within the total cell population. This could partly explain the decreased FoxP3 gene expression in the total cell population. Considering the increased expression of IL-1β genes, we speculate that cell handling and inadvertent cell stimulation could be a cause in our study. As presented here, there are disparities in the literature regarding the effect of cryopreservation on PBMCs. Several factors, including interexperimental differences, such as the use of different concentrated DMSO solutions, different surface staining antibody clones, and variations in the cell-handling protocol, may contribute to different results^38^. To standardize our methods, we employed standard of practice protocols and/or manufacturer instructions and attempted to mitigate confounders by performing the experiments in most of the cases, with two operators working side by side to support the cell-handling process. The latter can however also introduce individual variations in cell handling.

In our study setting, we used enriched PBMCs from healthy volunteers, and the limited number of experiments was repeated. To answer the clinically relevant questions, whether cryopreserved Tregs can potentially be useful for future immunotherapies or whether cryopreserved PBMCs can be used to generate immunomodulatory cells, and how the RBC lysate affect the clinical outcomes, it would be necessary to use patient blood in a larger scale using cryobags instead of cryovials as was used in this study. We investigated several different aspects of PBMCs for the effects of post-cryopreservation and ACK treatment. In the next step, we would arguably focus on a few assays, such as Treg suppressive functional assays and cell compositions, and expand the number of buffy coats to solidify the results and try to implement this in clinically relevant settings.

In summary, our results suggest that the phenotype and functionality of PBMCs are largely maintained after cryopreservation. Despite a lowered cell yield, as well as slight alterations in PBMC composition, the functionality remained intact; thus, we consider that cryopreservation of PBMCs in tolerance studies is feasible and safe *in vitro*. Treg composition and suppressive capacity were not altered. Downsides include a lowered cell yield as well as slight alterations in PBMC composition, although the functionality was intact. The upside of cryopreservation is mainly in the logistical part where it can potentially decrease the threshold to perform complex multi-center tolerance/immunotherapy studies in the future.

## Conclusions

ACK treatment mostly affects RBCs, while leukocyte subsets remain relatively unchanged. ACK treatment seems to improve the antigen sensitivity of memory T cells.

Cryopreservation reduces cell numbers and viability and seems to affect PBMCs on a gene expression level. The PBMC phenotype generally remains intact, though we noted a reduction of the CD4^+^ T-cell population. The Treg phenotype and suppressive functionality remain intact after cryopreservation. In conclusion, ACK treatment and cryopreservation of cell products appear to be feasible solutions to overcome logistically challenging tolerance-induction protocols.

## Supplemental Material

sj-docx-1-cll-10.1177_09636897251382315 – Supplemental material for Phenotypic and functional comparisons between cryopreserved and freshly isolated peripheral blood mononuclear cells with or without red blood cell lysate (ACK) treatment with special focus on regulatory T cells Supplemental material, sj-docx-1-cll-10.1177_09636897251382315 for Phenotypic and functional comparisons between cryopreserved and freshly isolated peripheral blood mononuclear cells with or without red blood cell lysate (ACK) treatment with special focus on regulatory T cells by Keyvan Habibi, Nils Ågren, Kaoru Okada, Heléne Johansson, Ming Yao and Makiko Kumagai-Braesch in Cell Transplantation
