# Supplementary material for: Phenotypic and functional comparisons between cryopreserved and freshly isolated peripheral blood mononuclear cells with or without red blood cell lysate (ACK) treatment with special focus on regulatory T cells
Source: Cell Transplant. 2025 Oct 29;34:09636897251382315. doi: 10.1177/09636897251382315 (PMC12576105; doi:10.1177/09636897251382315)
Supplement: sj-docx-1-cll-10.1177_09636897251382315 – Supplemental material for Phenotypic and functional comparisons between cryopreserved and freshly isolated peripheral blood mononuclear cells with or without red blood cell lysate (ACK) treatment with special focus on regulatory T cells [file sj-docx-1-cll-10.1177_09636897251382315.docx]

**Supplemental data:** Comparison of the geometric mean of cell proportions between the freshly enriched PBMCs vs the frozen PBMCs. Note that contamination of RBCs and granulocytes still exists after enrichment of PBMCs, please refer to the *Cell Separation* section of Material and Methods for the reasoning.

| Mean ± SD | Fresh | Frozen | P-value |
| --- | --- | --- | --- |
| Leukocytes ^a)^ | 68,89 ± 25,24 | 91,93 ± 7,314 | 0,0078* |
| Live CD45^+^ cells ^b)^ | 98,35 ± 1,403 | 94,7 ± 3,020 | 0,0078* |
| RBCs ^a)^ | 27,87 ± 22,72 | 7,094 ± 6,572 | 0,0078* |
| PBMCs ^c)^ | 83,38 ± 18,42 | 98,03 ± 1,820 | 0,0156* |
| Granulocytes ^c)^ | 13,52 ± 16,54 | 1,049 ± 1,505 | 0,0078* |
| Lymphocytes ^d)^ | 86,03 ± 11,35 | 84,31 ± 7,738 | 0,8438 |
| Monocytes ^d)^ | 13,84 ± 11,27 | 15,56 ± 7,695 | 0,8438 |
| NK cells ^e)^ | 14,13 ± 9,294 | 15,93 ± 9,050 | 0,0234* |
| B cells ^e)^ | 5,748 ± 4,460 | 7,655 ± 3,561 | 0,1562 |
| T cells ^e)^ | 75,51 ± 10,93 | 69,58 ± 12,67 | 0,0078* |
| CD4^+^ T cells ^f)^ | 66,31 ± 12,12 | 59,95 ± 14,26 | 0,0078* |
| Treg cells ^g)^ | 4,263 ± 2,611 | 2,941 ± 1,972 | 0,3750 |
| CD8^+^ T cells ^f)^ | 29,05 ± 11,43 | 32,14 ± 10,86 | 0,0234* |

The mean and standard deviation (SD) with corresponding p-values are shown (n=8). These cells were not treated with ACK.

a) % of all single cells (see Figure 1A), b) % of leukocyte gate, c) % in live CD45^+^ cells, d) % in PBMC gate, e) % in lymphocyte gate, f) % in CD3^+^ T cell gate and g) % in CD4^+^ T cell gate.

* Asterisk denotes a statistically significant result.
